# A novel bispecific aptamer targeting LAG3 and HER2 enhances T cell-mediated immunotherapy against HER2-positive cancer cells

**DOI:** 10.3389/fimmu.2025.1557910

**Published:** 2025-07-21

**Authors:** Rui Guo, Xiaoyang Chen, Lu Ying, Wenjing Zhang, Zufa Sabeel, Lianhui Zhao, Jian Dong, Yiyang Dong, Zhao Yang

**Affiliations:** College of Life Science and Technology, State Key Laboratory of Green Biomanufacturing, Innovation Center of Molecular Diagnostics, Beijing University of Chemical Technology, Beijing, China

**Keywords:** tumor, immunotherapy, bispecific aptamer, LAG3, HER2

## Abstract

**Objective:**

Malignant tumors are one of the leading causes of human death worldwide. In recent years, immunotherapy has become an emerging treatment method following surgery, radiotherapy, and chemotherapy. The study focused on two critical targets: human epidermal growth factor receptor 2 (HER2), a well-established tumor biomarker overexpressed in malignancies such as non-small cell lung cancer and hepatocellular carcinoma, and lymphocyte activation gene 3 (LAG3), an immune checkpoint molecule predominantly expressed on activated T lymphocytes and natural killer cells. Herein, we developed a novel bispecific aptamer (HLB-apt) to investigate its dual-targeting therapeutic potential in regulating tumor-immune cell interactions.

**Method:**

Firstly, the Moe algorithm was used to simulate the docking results between the aptamer and the corresponding protein. Then, the constructed HLB-apt was validated. In addition, based on A549, HepG2 cells and Jurkat cells, the functions and mechanisms of HLB-apt acting simultaneously with A549, HepG2 cells and Jurkat cells were verified through *in vivo and in vitro* experiments.

**Results:**

HLB-apt demonstrated specific binding capacity to both HER2-expressing tumor cells (A549 and HepG2) and LAG3-positive Jurkat cells. Notably, HLB-apt enhanced the killing effect of Jurkat cells on A549 and HepG2 cancer cells, with a killing rate of up to 29.00% for A549 and 7.46% for HepG2. Mechanistically, HLB-apt promotes the expression and secretion of IL-2, TNF-α, and granzyme B in activated Jurkat cells, and increases the expression of BAK1, BIM, and BAX in A549 and HepG2 cells. More importantly, HLB-apt significantly inhibited tumor growth in A549 and HepG2 tumor bearing mice and H&E staining revealed no overt histopathological abnormalities in major organs.

**Conclusion:**

Our findings demonstrate that HLB-apt exerts dual antitumor effects likely through simultaneously targeting LAG3 on T cells and HER2 on tumor cells, facilitating T cell recruitment and potentially interfering with immune checkpoint pathways. Therapeutic efficacy was markedly enhanced in tumor-bearing mice receiving combined HLB-apt and Jurkat cell administration, This HLB-apt holds promising clinical potential for malignancies characterized by HER2 overexpression.

## Introduction

Globally, cancer remains a leading cause of mortality and represents a substantial economic burden (1, 2). According to the latest estimates from the International Agency for Research on Cancer (IARC), there were nearly 20 million new cancer cases and 9.7 million cancer deaths worldwide in 2022 (3). Surgical resection has satisfactory results for early solid cancer, but it is difficult to remove the cancer lesion in the patients with advanced or metastatic cancers and hematological malignancies (4, 5). Chemoradiotherapy and targeted therapy can shrink cancers and eliminate small metastases in the body, reducing the risk of recurrence and metastasis. However, the drug-resistance and side effects, such as nausea, vomiting and hair loss, caused by chemoradiotherapy and targeted therapy should not to be ignored (6). Therefore, it is urgent to develop novel anti-cancer strategies to reduce mortality and improve the living quality of patients. Recently, immunotherapy has become the fourth pillar of cancer treatment after surgery, chemoradiotherapy and targeted therapy, which fights cancer by generating or enhancing immune responses against cancer cells.

Aptamers are single-stranded oligonucleotides (25–80 nucleotides in length) characterized by well-defined structural motifs through intramolecular base pairing. Their folding plasticity enables the formation of diverse secondary structures, including stems, loops, bulges, pseudoknots, G-quadruplexes, and kissing hairpins (7). These structural elements cooperatively assemble into unique three-dimensional conformations, which confer high-affinity molecular recognition of specific targets through shape complementarity and electrostatic interactions (8). Therefore, aptamers are excellent components of targeted systems for drug delivery and are typically used as materials for targeted therapy (9). Bispecific aptamers can simultaneously bind two different cell surface receptors on two different cells, such as cancer cells and lymphocytes, inducing enhanced anti-cancer immunity by targeting both cancer cells and lymphocytes simultaneously (10). In recent years, aptamer engineering has shown significant potential for application in the field of tumor immunotherapy. For instance, Zheng et al. (11) innovatively constructed a stable aptamer structure based on a Y-shaped DNA scaffold, which specifically targets natural killer cells (NK cells) in the tumor microenvironment through spatially directed regulation mechanisms, significantly improving the solid tumor infiltration efficiency of adoptive immunotherapy for hepatocellular carcinoma. In a similar research strategy, aptamer based CD16/programmed death ligand 1 (PD-L1) bispecific molecules have been successfully constructed, which exhibit significant anti-tumor synergistic effects through a dual mechanism of synergistic activation of NK cell recruitment and PD-L1 immune checkpoint blockade (12). Notably, the PD-L1/programmed cell death protein 1 (PD-1) bifunctional aptamer Ap3-7c, created by the Sun team (13), forms a stable covalent coupling complex following site modification with dibenzocyclooctyne. Its pioneering “recognition before binding” mechanism competitively inhibits the PD-1/PD-L1 signaling axis while achieving sustained target occupancy. Research indicates that this strategy can elicit robust anti-tumor immune responses.

Lymphocyte activating gene 3 (LAG3) is a potential cancer immunotherapy target located near CD6 on human chromosome 4, expressing on activated human T and NK cells (14). Currently, many experimental therapeutic agents targeting LAG3 are being tested in clinical trials of human cancer. The first of its kind was IMP321, which is a 200 kDa soluble chimeric recombinant fusion protein. In clinical trials, IMP321 successfully treated renal cell carcinoma, metastatic breast cancer and melanoma in some patients (15). Moreover, in a completed phase I trial of metastatic breast cancer, the initial efficacy of IMP321 combined with chemotherapy drug paclitaxel showed an objective response rate(ORR)of 50%, while ORR with paclitaxel alone was only 25% (16). Relatlimab is the first LAG3 monoclonal antibody under development. In 2017, the annual conference of the American Society of Clinical Oncology presented the efficacy of relatlimab and nivolumab in melanoma patients after immunotherapy (17). The ORR of 31 melanoma patients was 16%, and the duration of response (DOR) was 45%. The therapeutic effect appears encouraging while safety remains acceptable. On March 18, 2022, the FDA approved the combination of relatlimab (anti-LAG3) and nivolumab (anti-PD-1) for unresectable or metastatic melanoma, marking LAG3 as the third validated immune checkpoint target after CTLA-4 and PD-1 (18). Therefore, LAG3 is a promising immunotherapy target.

Human epidermal growth factor receptor 2 (HER2) is a tyrosine kinase receptor with a molecular weight of 185 kDa, which is overexpressed in breast cancer, gastric cancer, ovarian cancer, lung cancer and liver cancer (19–23). Trastuzumab is the first HER2 monoclonal antibody approved by FDA (24), and has been used for the treatment of metastatic HER2 positive breast cancer for more than ten years (25). Later, in a successful clinical trial, FDA approved the combination of trastuzumab and docetaxel to treat metastatic breast cancer (26), which can improve the survival endpoint and quality of life of patients with advanced HER2 positive breast cancer who receive chemotherapy. In addition, the combination of trastuzumab and other drugs was associated with longer disease-free progression (median, 7.4 months vs 4.6 months; *P*<0.001), longer survival (median survival, 25.1 months vs 20.3 months; *P*<0.01), lower 1-year mortality rate (22% vs 33%; *P*<0.008), and higher objective response rate (50% vs 32%; *P*<0.001) (27). In summary, HER2 plays a crucial role in cell migration, proliferation, survival, angiogenesis, and metastasis through various intracellular signaling cascades, making it a promising target in cancer treatment (28, 29).

**Figure 1:**
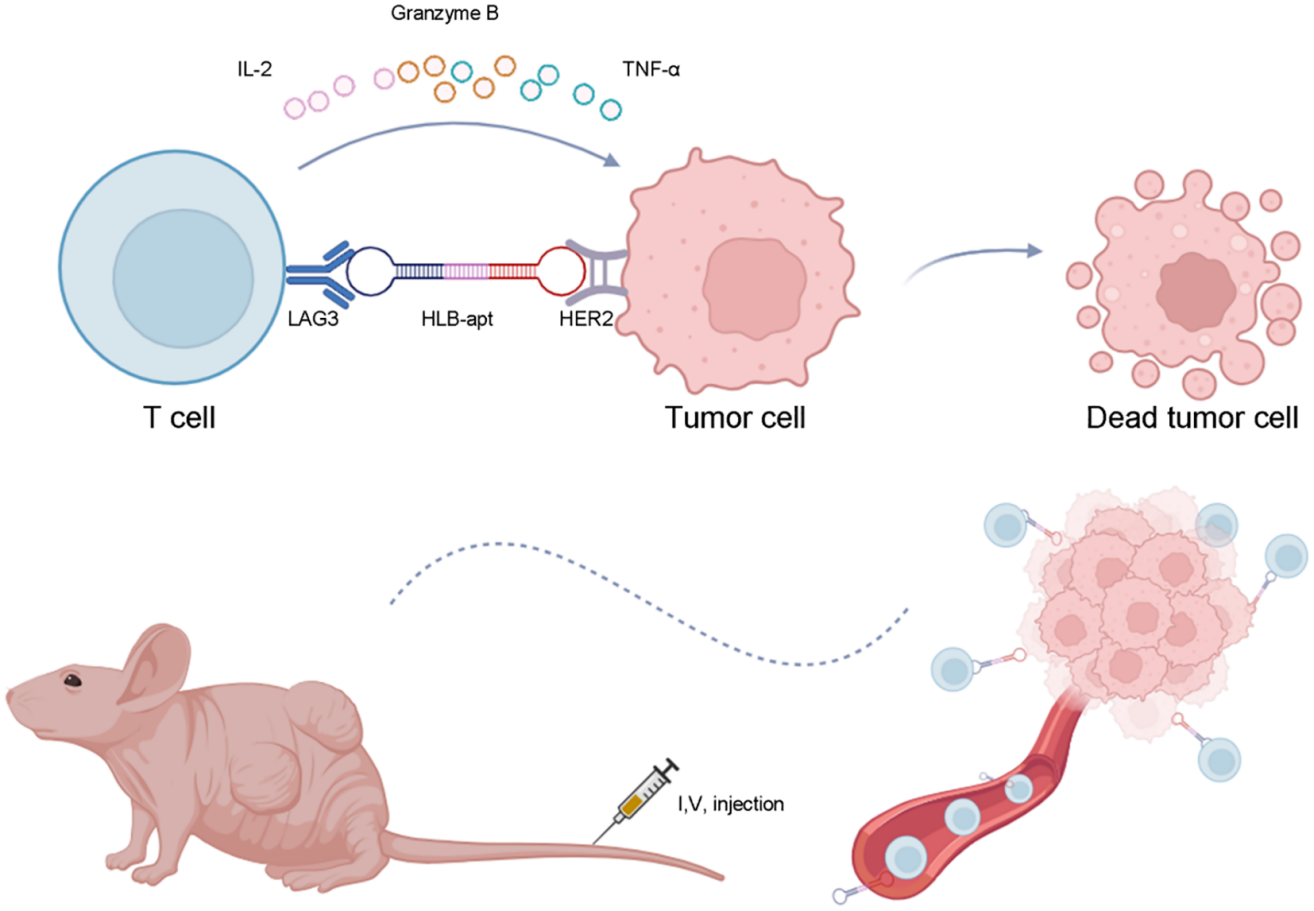
Schematic illustration of anticancer immunotherapy of HLB-apt.

In this study, we constructed a bivalent bispecific aptamer (HLB-apt) targeting LAG3 and HER2 simultaneously, which recruits T cells to HER2-expressing cancer cells, thereby enhancing the anti-cancer immune response *in vitro* and *in vivo* (**Figure 1**).

## Materials and methods

### Preparation of HLB-apt

Simulate the binding of LAG3-apt to LAG3 protein, HER2-apt to HER2 protein, and HLB-apt to LAG3 protein and HER2 protein using Moe algorithm. HLB-apt is composed of LAG3-apt and HER2-apt. It uses 10 base T as the linker, the sequence is GGGAGAGAGATATAAGGCCTTTTTTTTAGCCGGGGGGGGGGGGGGGGGGGGGGCGCGT, which is single stranded DNA. HLB-apt was prepared by Biotechnology (Shanghai) Co., Ltd. via solid-phase synthesis and purified by reverse phase HPLC (purity > 95%).

### Polyacrylamide gel electrophoresis

The successful synthesis of HLB-apt was evaluated by denatured 10% polyacrylamide gel electrophoresis (containing 7 M urea). Electrophoresis was performed in TBE buffer at a voltage of 200 V, followed by staining with Gel Red (Thermo Fisher Scientific) and visualization. In terms of stability determination, 50% FBS (Thermo Fisher Scientific) and 500nM HLB-apt was incubated at 56°C for different times (0 h, 6 h, 12 h, 24 h and 48 h), and then the integrity of the aptamer was analyzed by denatured 10% polyacrylamide gel electrophoresis.

### Cell culture

Human lung cancer cell lines (A549), human liver cancer cell lines (HepG2), and human T-cell leukemia cells (Jurkat) were cultured in 1640 medium supplemented with 10% FBS (PAN Biotech, Germany) and 1% streptomycin/penicillin (Thermo Fisher Scientific), and incubated at 37°C in a 5% CO_2_ incubator. A549 and HepG2 cells use ≤ 20 passages, Jurkat cells use ≤ 15 passages. All cell lines were regularly validated through STR typing (A549) and flow cytometry (Jurkat CD3 +> 95%).

### Cell binding

Cultivate A549 and HepG2 cells (4×10^5^), suspend cells in 500 μL PBS. Add 500 nM cy5-HER2-apt. Gently shake and incubate for 30 min. Centrifuge and discard the supernatant. Wash twice with PBS and analyze by flow cytometry. Cultivate Jurkat cells (4×10^5^), suspend cells in 500 μL PBS. Add 500nM cy3-LAG3-apt to, gently shake and incubate for 30 min, centrifuge, discard the supernatant, wash twice with PBS, and analyze by flow cytometry. Repeat the experiment three times in combination with the measurement.

### Competitive binding

The HER2 antibody was purchased from abcam and is a monoclonal antibody with clone number EP2324Y and antigenic epitope ab108371. LAG3 antibody was purchased from Bioss and is a polyclonal antibody with an antigenic epitope of bs-2646R. Cultivate A549 and HepG2 cells (4×10^5^), suspended at 500 μL in PBS. Simultaneously add 500 nM cy5-HER2-apt. and HER2 antibody. Similarly, add 500 nM cy5-HER2-apt. and HLB-apt at the same time, gently shake and incubate for 30 min. Centrifuge and discard the supernatant. Wash twice with PBS and analyze by flow cytometry. Cultivate Jurkat cells (4×10^5^), suspended at 500 μL in PBS. Simultaneously add 500 nM cy3-LAG3-apt and LAG3 antibodies. Similarly, 500 nM cy3-LAG3-apt and HLB-apt were added simultaneously, centrifuged, the supernatant was discarded, washed twice with PBS, and analyzed by flow cytometry. Repeat the experiment three times based on the measurement results.

### The specificity of HLB-apt

Inoculate A549 and HepG2 (4×10^5^) in a six well plate for 12 h, then add equimolar (500nM) fluorescent labeled aptamers (cy3-LAG3-apt, cy5-HER2-apt., HLB-apt) and incubate at 37°C for 30 min. Use cells without added aptamers as control. After washing three times with PBS, observe and take photos using a fluorescence microscope. In addition, Jurkat cells (1×10^6^) incubate with equimolar (500 nM) fluorescent labeled aptamers (cy3-LAG3-apt, cy5-HER2-apt, HLB-apt) at 37°C for 30 min. Then, mix the cell mixture with 800×g Centrifuge to remove unbound aptamers. After washing with PBS, resuspend Jurkat cells at 500 μL in PBS and observe and take photos using a fluorescence microscope on a six well plate. Finally, use the matching imaging processing software to obtain signal strength.

### Recruitment of Jurkat cells

Labeling cancer cells with hydroxyfluorescein diacetate succinimide lipid (CFSE) (A549 and HepG2, 4×10^5^) for 10 min, eFluro670 labeled Jurkat cells (1×10^6^) for 10 min, Wash three times with PBS, and resuspend the cells in PBS. Mix cancer cells and Jurkat cells separately in a six well plate, and add equal moles (500nM) of different aptamers (LAG3-apt, HER2-apt., LAG3-apt/HER2-apt, HLB-apt). Incubate at 37°C for 1 h. Afterwards, wash three times with PBS to remove any unattached Jurkat cells, and take photos under a fluorescence microscope for observation.

### CCK8 assay

A549 and HepG2 cells were inoculated into a 96 well plate using the CCK-8 assay kit (C0037, Beyotime, China). 500nM HER2-apt and 500nM HLB-apt were added to determine cell viability. At 24 and 48 h of cell culture, extract the culture medium from each well and discard it. Add 100 μL to each well containing 10% CCK-8 culture medium. Then transfer to a 5% CO_2_ incubator and incubate for 2 h. Measure the absorbance at 450 nm on the Multiscan FC microplate reader (51119080, Thermo Fisher Scientific, USA). Using the GraphPad Prism 8, perform statistical checks on the experimental results. The calculation of cell viability is as follows: Cell viability = OD (experimental group)/OD (control group) × 100%.

### Quantitative real-time PCR

The RNAprep pure cell Kit (DP430, Tiangen, China) was used to extract total RNA from A549, HepG2 and Jurkat cells. To do reverse transcription, the RNA was reverse-transcribed into cDNA using the FastKing RT Kit (KR116-02, Tiangen, China) in conjunction with a Real-Time PCR System (QuantStudio1-A40425, Thermo Fisher Scientific, USA) and PowerUp SYBR Green Master Mix (01000439, Thermo Fisher Scientific, USA). Ct (cycle threshold) was calculated and normalized to GAPDH. Apply the 2^−ΔΔCt^ technique. **Table 1** shows the primers purchased from Tsingke Biotechnology Co.,Ltd.

**Table 1:** The sequences and docking scores of LAG3-apt, HER2-apt and HLB-apt.

Aptamer	Sequence	Docking score
LAG3-apt 1#	GGGAGAGAGATATAAGGGCCTCCTGATACCCGCTGCTATCTGGACCGATCCCATTACCAAATTCTCTCCC (30)	-95.1786 kcal/mol
LAG3-apt 2#	GGGAGAGAGAUAUAAGGGUCUUGCCUGAUGGAGUUGGAGAGGACCAUCACCCAUUACCAAAUUCUCUCCC (30)	/
LAG3-apt 3#	GGGAGAGAGAUAUAAGGGACCGAAUCUGGUUAAUCUGCCUCACGUUAGGCCCAUUACCAAAUUCUCUCCC (30)	/
HER2-apt 1#	ATACCAGCTTATTCAATT-N40-AGATAGTAAGTGCAATCT (31)	/
HER2-apt 2#	AGCCGCGAGGGGAGGGATAGGGTAGGGCGCGGCT (32)	-69.3342 kcal/mol
HLB-apt	GGGAGAGAGATATAAGGGCCTCCTGATACCCGCTGCTATCTGGACCGATCCCATTACCAAATTCTCTCCCTTTTTTTTTTAGCCGCGAGGGGAGGGATAGGGTAGGGCGCGGCT	-118.1522 kcal/mol

### Western blot

Use BCA reagent (PC0020, Solarbio, China) to evaluate the total protein content. Then, the proteins were separated using SDS-PAGE and transferred onto a PVDF membrane (Millipore, USA). After 1 h of shaking in TBST containing 5% skim milk, the following monoclonal antibodies were added: anti-human β-actin (1:5000; Cell Signalling Technology, USA), anti-BAK1 (1:1000; Cell Signaling Technology, USA), anti-BIM (1:1000; Cell Signaling Technology, USA), and anti-BAX (1:1000; Cell Signaling Technology, USA). After 1 hour of incubation with a secondary antibody and horseradish peroxidase (HRP) at 25°C, the membrane was washed three times for 20 min each with 1×TBST buffer. We observed the proteins using an Enhanced Chemiluminescence (ECL) kit (PE0010, Solarbio, China) and then analyzed the pictures using ImageJ. The experiment was repeated three times.

### CFSE measurement of Jurkat cell proliferation

Jurkat cells were stained with CFSE working solution for 20 min. Centrifuge, wash three times with PBS, count with a cell counting plate to obtain 5×10^6^ cells per well in a six well plate, and divide into control group, LAG3-apt group and HLB-apt group, 500 nM aptamer was added to each well, and incubated in the incubator for 48 h, and then detected by flow cytometry.

### *In vitro* killing experiment

CFSE working solution was added into A549 and HepG2 cells, stained in the incubator for 15 min, washed with PBS for 3 times, and counted by cell counting plate. The cell concentration was 2×10^5^/mL. The effect-to-target ratio of A549 group and HepG2 group was 5:1 and 2:1 respectively. After adding Jurkat cells, the cells were divided into control group, Jurkat group, LAG3-apt group, HER2-apt group, LAG3-apt/HER2-apt group and HLB-apt group, which were incubated in the incubator for 24 h. The supernatant and adherent cells were collected, and the PI working fluid was added for 15 min, and then washed three times with PBS, and then suspended with 500 μL PBS, and then detected by flow cytometry.

### Intracellular cytokine analysis by flow cytometry

Jurkat cells and cancer cells (A549 and HepG2 cells) were seeded in a 1:1 ratio into a 24-well plates, and then incubated with 500nM aptamer at 37°C for 24 h, which were divided into Control group, Jurkat group, LAG3-apt group, HER2-apt group, LAG3-apt/HER2-apt group and HLB-apt group. The supernatant was collected by centrifugation, and the contents of granzyme B (Gzms B), interleukin-2 (IL-2) and tumor necrosis factor-α (TNF-α) were detected by corresponding ELISA kit.

### Establishment of A549 and HepG2 nude mouse transplanted cancer model

Sixty-four 4-week-old Balb/c female nude mice were purchased from Vital River Company and divided into two groups, and 1×10^7^ A549 and HepG2 cells were injected subcutrically into the armpits of the mice, respectively. Sample size was determined based on: power analysis (α=0.05, β=0.2) using G*Power 3.1, with effect size estimated from pilot data (40% tumor inhibition ± 25% SD).

When the tumor size was measured to be about 50 mm^3^, 40 mice were divided into the following 5 groups: PBS group, Jurkat cell group, Jurkat cell + LAG3-apt group, Jurkat cell + HER2-apt group and Jurkat cell + HLB-apt group were injected with 10 nM Jurkat cells containing different aptamers through the tail vein (PMA/Ion activated, 5×10^6^) was injected every three days, while the control group was injected with the same amount of PBS every three days. During this period, the changes of mouse cancers were observed, the length (x) and width (y) of mice were recorded, and the volume (V) of mouse cancers was calculated: V=(x^y^2^)/2. When the tumor volume of mice in PBS group grew to 1000 mm^3^, the mice were killed, the tumors were removed and photographed, and the hearts, livers, spleens, lungs and kidneys of mice were dissected and stored in 4% paraformaldehyde.

### Three-dimensional quantitative imaging system fluorescence photography

24 mice were divided into two groups with 12 mice in each group. When the tumor grew to about 50 mm^3^, 10 nM fluorescently labeled aptamer was injected through the tail vein and divided into PBS group, LAG3-apt group with cy5 fluorescence label, HER2-apt group with cy5 fluorescence label and HLB-apt group with cy7 fluorescence label. 24 h later, major organs (heart, liver, spleen, lung, kidney) and tumors were dissected, and fluorescence photographs were taken by three-dimensional quantitative imaging system. The fluorescence intensity of each tissue was quantified by ImageJ.

### H&E dyeing

The tissue samples were fixed with 4% paraformaldehyde to prepare slices of the tissue samples, which were soaked in an appropriate buffer to adjust the pH value before staining. First, hematoxylin is used, the nucleus is stained, and then the cytoplasm is stained with eosin. After hematoxylin staining, the hematoxylin semen should be washed with running water, then treated with 1% hydrochloric alcohol for 3 s, washed for 30 s, then dyed with 0.5% eosin solution for 3 min, washed for 2 s. A range of alcohol concentrations are used to dehydrate and transparent agents such as phenol and xylene are used. After transparency, the slices will become clearer and easier to stain. It is sealed with a neutral resin to prevent future fading and ensure that the sections remain clear when viewed under a microscope. Finally, they were observed and photographed under a fluorescence microscope.

### Immunohistochemical

Store mouse tumors in formaldehyde at 4°C and send them to Servicebio (Wuhan, China) for immunohistochemistry. Afterwards, observe and take photos under a fluorescence microscope.

### Statistical analysis

Statistical evaluations in this investigation were performed utilizing GraphPad Prism software (version 8.0). Quantitative outcomes derived from triplicate biological replicates were expressed as arithmetic mean ± standard deviation (SD). Intergroup comparisons were analyzed through Student’s t-test (two-group comparisons) or one-way ANOVA (multi-group comparisons), with all hypothesis tests executed in a two-tailed framework. A predetermined significance threshold of *P<0.05 was implemented for all inferential analyses.

## Results

### Preparation and characterization of HLB-apt

**Figure 2:**
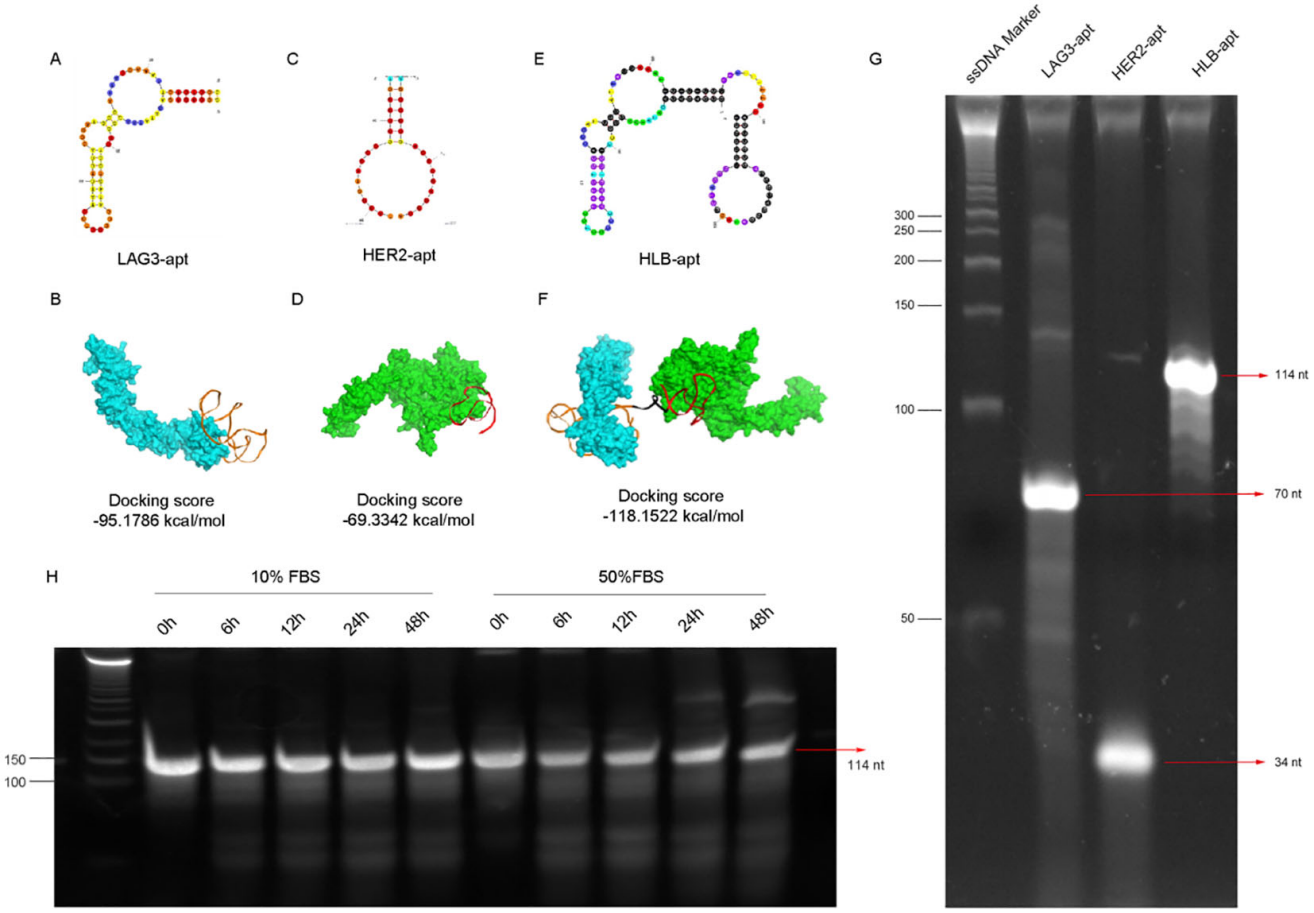
The design, structure and characterization of HLB-apt. **(A)** Structure of LAG3-apt. **(B)** The binding of LAG3-apt and LAG3 protein. **(C)** Structure of HER2-apt. **(D)** The binding of HER2-apt and HER2 protein. **(E)** Structure of HLB-apt. **(F)** HLB-apt combines LAG3 protein and HER2 protein simultaneously. **(G)** Polyacrylamide gel electrophoresis of LAG3-apt, HER2-apt and HLB-apt. **(H)** The stability analysis of HLB-apt in 10% FBS or 50% FBS at 0 h, 6 h, 12 h, 24 h and 48 h.

In order to construct a stable HLB-apt, we selected the apatamer sequences of LAG3 and HER2 according to previous reports (30–32), which were summarized in **Table 1**. The Moe algorithm is used to simulate the docking results between the aptamers and the corresponding protein. The docking results showed that the docking score between LAG3-apt and LAG3 protein was -95.1786 kcal/mol, and the docking score between HER2-apt and HER2 protein was -69.3342 kcal/mol. The docking results showed that LAG3-apt 1# and HER2-apt 2# were the best (**Figures 2A–D**). In addition, ten bases thymine (T) was used as a linker to construct a semi closed “gate” structure between LAG3-apt and HER2-apt. Subsequently, HLB-apt (**Figure 2E**) and simultaneous docking with LAG3 protein and HER2 protein (**Figure 2F**) were performed, and the results showed a docking score of -118.1522 kcal/mol, indicating that HLB-apt is relatively stable, as expected.

To verify whether HLB-apt was successfully constructed, it was characterized by polyacrylamide gel electrophoresis. As shown in **Figure 2G**, the target stripe of LAG3-apt is 70 nt, that of HER2-apt is 34 nt, and that of HLB-apt is 114nt. Subsequently, we validated the stability of HLB-apt by incubating it with 10% and 50% serum at 56°C. After 48 h, HLB-apt incubated with 10% serum showed little degradation, while HLB-apt incubated with 50% serum remained strong signal (**Figure 2H**). The above results indicate that HLB-apt has been successfully constructed and has a certain degree of stability.

### Bindings of HLB-apt and monovalent aptamers to cancer or Jurkat cells

**Figure 3:**
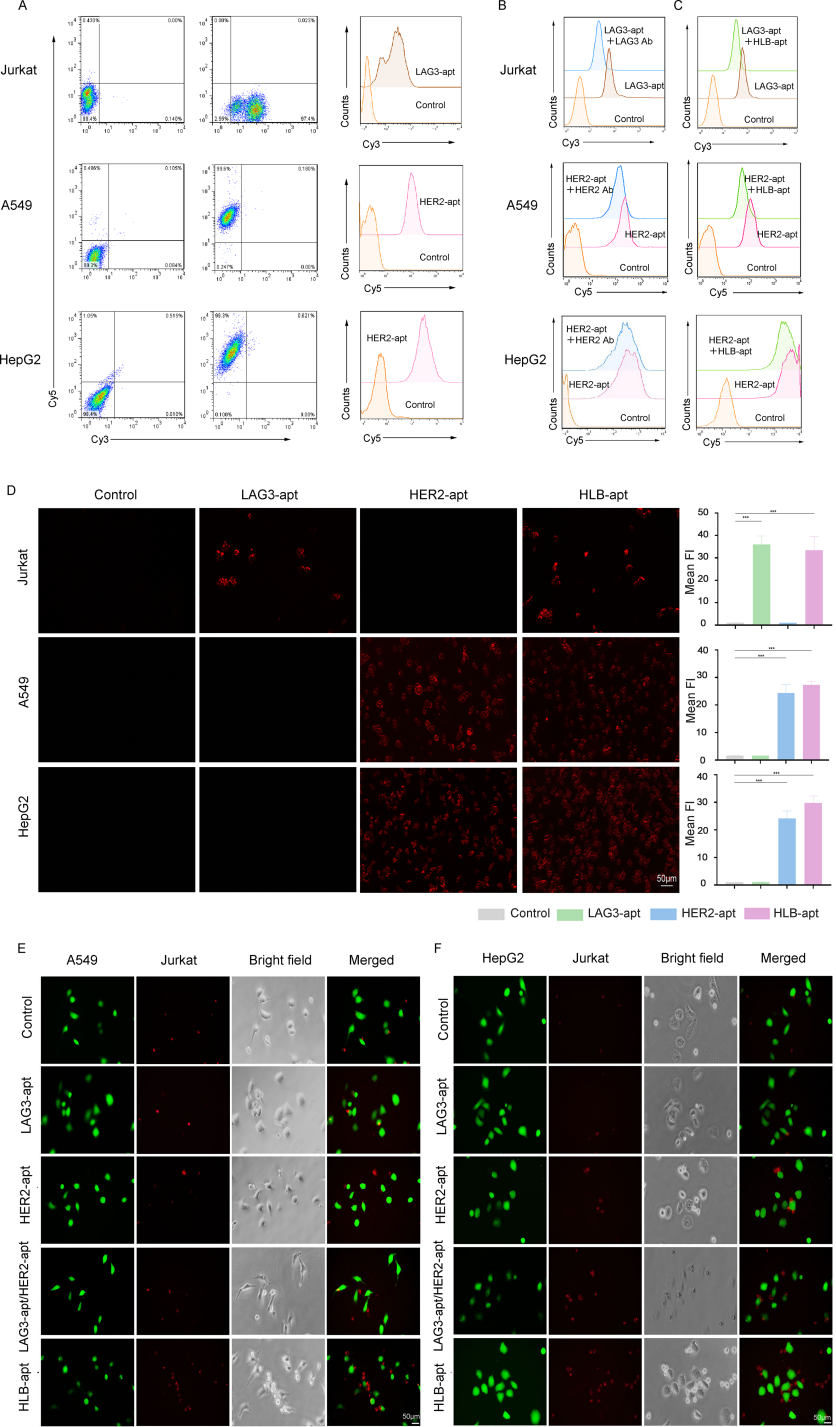
HLB-apt recognizes and recruits Jurkat cells to cancer cells. **(A)** The binding abilities of LAG3-apt and HER2-apt to Jurkat cells or cancer cells were detected by flow cytometry. **(B)** Competitive binding assays of LAG3-apt and anti-LAG3 antibody in Jurkat cells, and HER2-apt and anti-HER2 antibody in cancer cells. **(C)** Competitive binding assays of LAG3-apt and HLB-apt in Jurkat cells, and HER2-apt and HLB-apt in cancer cells. **(D)** Fluorescence microscopy images of Jurkat, A549, and HepG2 cells incubated with cy3 labeled LAG3-apt, cy5 labeled HER2-apt, and cy7 labeled HLB-apt. **(E, F)** HLB-apt promoted the recruitment of Jurkat cells (stained red with eFluor670) to A549 or HepG2 cells (stained green with CFSE). (Data analysis was conducted by student t-test (comparison between two groups) or one-way analysis of variance (comparison among multiple groups), and the significance levels were ***P<0.001.)

After construction, it is crucial to reassess the binding of HLB-apt to target cells. For this purpose, we incubated cy3 fluorescent labeled LAG3-apt with Jurkat cells, and cy5 fluorescent labeled HER2-apt with cancer cells. The results showed that LAG3-apt could bind to Jurkat cells, while HER2-apt could incubate with A549 and HepG2 cells (**Figure 3A**).

To further investigated the binding site of the HLB-apt and univalent aptamers, HLB-apt or the corresponding antibodies were added with fluorescent labeled univalent aptamers together into the tumor or Jurkat cells. The results showed that the addition of LAG3 antibody significantly decreased the binding of LAG3-apt to Jurkat cells (**Figure 3B**, upper panel). Similarly, the addition of HER2 antibody remarkably reduced the combination of HER2-apt to A549 and HepG2 cells (**Figure 3B**, middle and lower panels). In addition, compared to adding only LAG3-apt, the fluorescence intensity was significantly reduced when LAG3-apt and HLB-apt were added simultaneously. Similarly, compared to adding only HER2-apt, the fluorescence intensity was significantly reduced when both HER2 apt. and HLB-apt were added simultaneously (**Figure 3C**). Therefore, Competitive binding assays suggest that HLB-apt shares epitopes with LAG3/HER2 antibodies, indicating potential engagement with LAG3 on T cells.

Additionally, we evaluated whether HLB-apt can specifically recognize its corresponding target cells. To this end, the fluorescence images of A549, HepG2, and Jurkat cells incubated with cy3-LAG3-apt, cy5-HER2-pt, and cy7-HLB-apt were observed under a fluorescence microscope. Significant fluorescence signals were observed in A549 cells and HepG2 cells with high HER2 expression, and in Jurkat cells with high LAG3 expression. After the addition of HLB-apt, significant fluorescence signals were observed in both cancer cells and Jurkat cells (**Figure 3D**). These results confirm that the prepared HLB-apt can bind specifically to cancer cells and Jurkat cells through HER2-apt and LAG-apt, respectively.

To verify whether Jurkat cells and cancer cells can have better interactions in the presence of HLB-apt. We labeled CFSE cancer cells with green fluorescence and Jurkat cells with red fluorescence using efluor670. Then, they were co-incubated with different aptamers. In the presence of HLB-apt, A549 and HepG2 recruited more Jurkat cells, while no Jurkat cell recruitment was observed in the control group without an aptamer (**Figures 3E, F**). Therefore, we infer that HLB-apt can to some extent recruit more Jurkat cells around cancer cells, which may promote Jurkat cell killing of cancer cells.

### HLB-apt induced apoptosis of A549 and HepG2 cells by up-regulating the expression of BAK1, BAX and BIM proteins

**Figure 4:**
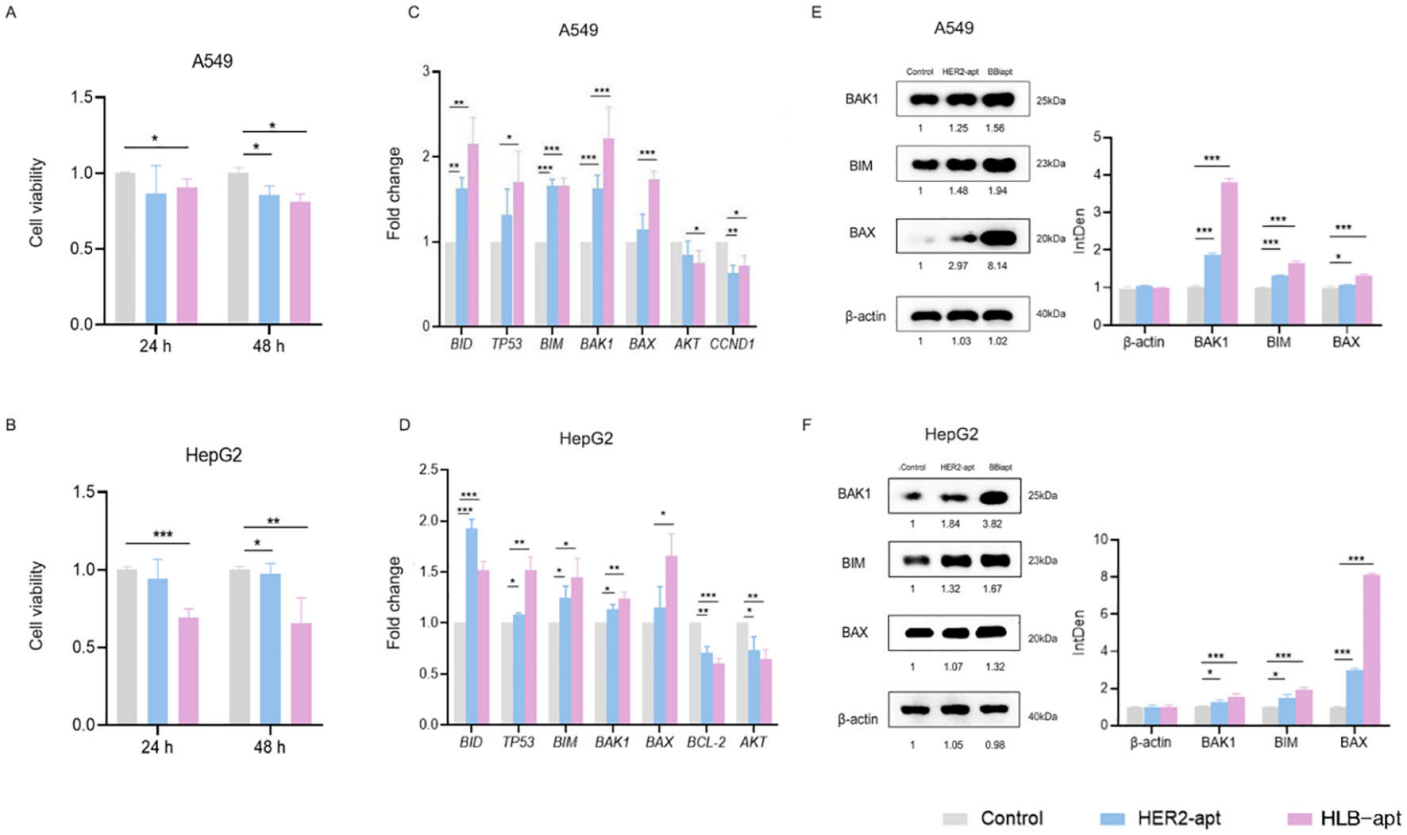
HLB-apt induces cell apoptosis by up-regulating the expression of BAK1, BIM, and BAX proteins in A549 and HepG2 cells. A549 **(A)** and HepG2 **(B)** cells were treated with 500 nM HLB-apt for 24 and 48 h. Using qPCR to detect the mRNA expression levels of apoptosis and cell cycle related genes in A549 **(C)** and HepG2 **(D)** cells, and treating A549 and HepG2 cells with 500 nM PEI. **(E, F)** Select genes that meet expectations from qPCR for Western blotting. Data represent mean of 3 determinations per condition repeated 3 times. (Data analysis was conducted by student t-test (comparison between two groups) or one-way analysis of variance (comparison among multiple groups), and the significance levels were *P<0.05, **P<0.01, and ***P<0.001).

Cancer cells have the characteristic of infinite proliferation. In order to evaluate the effect of HLB-apt on the proliferation ability of lung cancer and liver cancer cells, A549 and HepG2 cells were selected for this study. The changes in cell viability were analyzed through the CCK8 experiment, as shown in **Figures 4A, B**, the results showed that at 48 h, HER2-apt and HLB-apt could significantly inhibit the proliferation of A549 and HepG2 cells (*P*<0.01), and HLB-apt had a better inhibitory effect.

Subsequently, by studying the mechanism of action of HLB-apt on A549 and HepG2 cells, we discovered the mRNA expression of genes involved in cancer cell apoptosis, including *BID, TP53, BIM, BAK1, BAX, AKT, CCND1, AKT, and BCL-2.* Compared with the control group, the three apoptosis related genes *BAK1, BIM*, and *BAX* in A549 and HepG2 cells treated with HLB-apt were all expressed at significantly higher mRNA levels (*P*<0.01) (**Figures 4C, D**).

**Figure 5:**
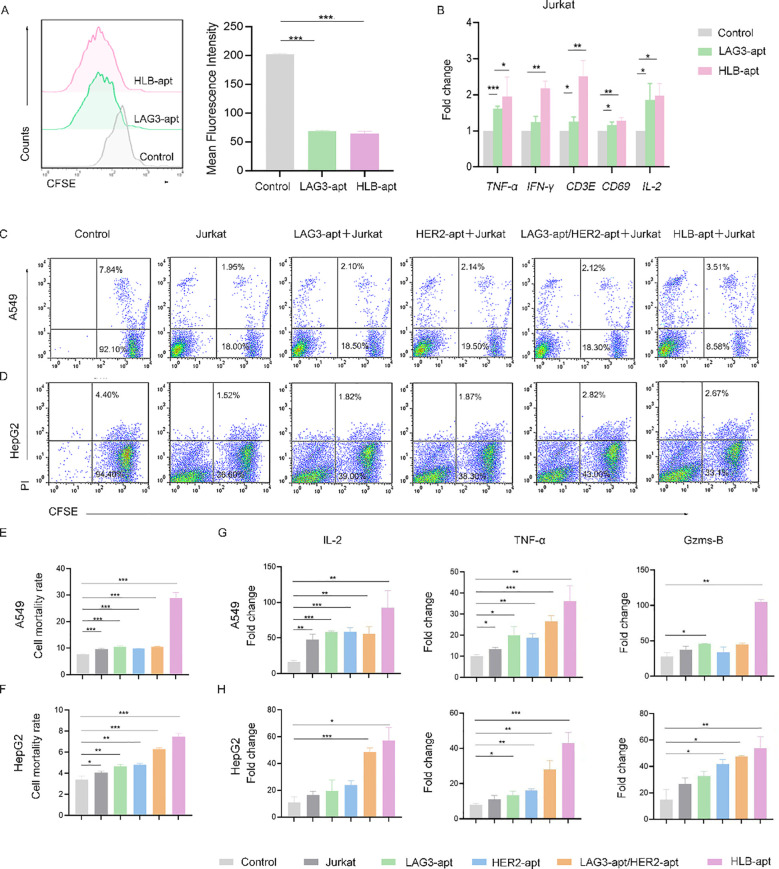
HLB-apt enhances the killing effect of Jurkat cells. **(A)** Detection of Jurkat cells proliferation after 48 h of treatment with different aptamers using CFSE. Application of qPCR to detect mRNA expression levels of cytokines in Jurkat **(B)** cells. Flow cytometry analysis of the cell activity of **(C)** A549 and **(D)** HepG2 in the presence of Jurkat cells and different aptamers. Mortality statistics of **(E)** A549 cells and **(F)** HepG2 cells. **(G)** When A549 cells were co cultured with Jurkat cells and LAG3-apt, HER2-apt, LAG3-apt/HER2 apt, and HLB-apt were added, ELISA was used to detect changes in the concentrations of IL-2, TNF - α, and Gzms-B. **(H)** When HepG2 cells were co cultured with Jurkat cells and LAG3-apt, HER2-apt, LAG3-apt/HER2 apt, and HLB-apt were added, ELISA was used to detect changes in the concentrations of IL-2, TNF - α, and Gzms-B. (Data analysis was conducted by student t-test (comparison between two groups) or one-way analysis of variance (comparison among multiple groups), and the significance levels were *P<0.05, **P<0.01, and ***P<0.001).

We further detected the expression of three apoptosis related proteins BAK1, BIM, and BAX in A549 and HepG2 cells. The results showed that compared with the control group, the expression of BAK1, BIM, and BAX proteins in the two cell lines treated with HLB-apt was significantly upregulated (*P*<0.01), in line with expectations (**Figures 5E, F**). More importantly, this is consistent with the results of qPCR. In summary, HLB-apt significantly upregulated the expression of *BAK1, BIM*, and *BAX* at the mRNA and protein levels.

### HLB-apt enhances the killing effect of Jurkat cells

Furthermore, we investigated the effect of HLB-apt treatment on Jurkat cells. We used CFSE staining to investigate the effect of HLB-apt on Jurkat cell proliferation. The results showed that LAG3-apt and HLB-apt treatment significantly improved Jurkat cell proliferation ability (*P*<0.001) (**Figure 5A**). Next, we used qPCR to detect the changes in cytokine expression levels of Jurkat cells after HLB-apt treatment. The results showed that after HLB-apt treatment, T cell activation markers (CD69, CD3E), interleukin (IL-2), and other cytokines in Jurkat cells were significantly overexpressed (*P*<0.05) (**Figure 5B**), indicating an increase in Jurkat cell killing ability against cancer cells. Thus improving the killing ability of Jurkat cells against cancers.

In order to verify whether the killing ability of Jurkat cells has been improved, we conducted *in vitro* cancer killing experiments. To this end, the cancer cells were co incubated with corresponding aptamers and Jurkat cells for 24 h. Afterwards, suspended cells and adherent cells in the culture medium were collected and stained with CFSE/propidium iodide (PI) for flow cytometry analysis. As shown in **Figure 5C**, A549 cells treated with Jurkat cells showed 9.77% apoptosis or necrosis, while the percentage of apoptotic and necrotic cancer cells increased to 29.00% after the addition of HLB-apt. Similarly, HepG2 cells treated with Jurkat cells showed 3.98% apoptosis or necrosis, and the percentage of apoptotic and necrotic cancer cells increased to 7.46% after the addition of HLB-apt (**Figures 5D–F**). The killing ability of Jurkat cells treated with HLB-apt on human lung cancer cell line A549 and human liver cancer cell line HepG2 was significantly improved. These results further demonstrate that HLB-apt can enhance the killing effect of Jurkat cells.

To further evaluate the killing effect of HLB-apt assisted Jurkat cells, ELISA was used to measure cytokine secretion of TNF-α, IL-2 and Gzms B. Both A549 cells and HepG2 cells showed a significant increase in the concentration of four cytokines in the culture medium when co incubated with Jurkat cells or when different aptamers were added simultaneously, especially when HLB-apt was added (**Figures 5G, H**). These results further indicate that HLB-apt can enhance the anti-cancer ability of Jurkat cells.

### Imaging distribution in mice

**Figure 6:**
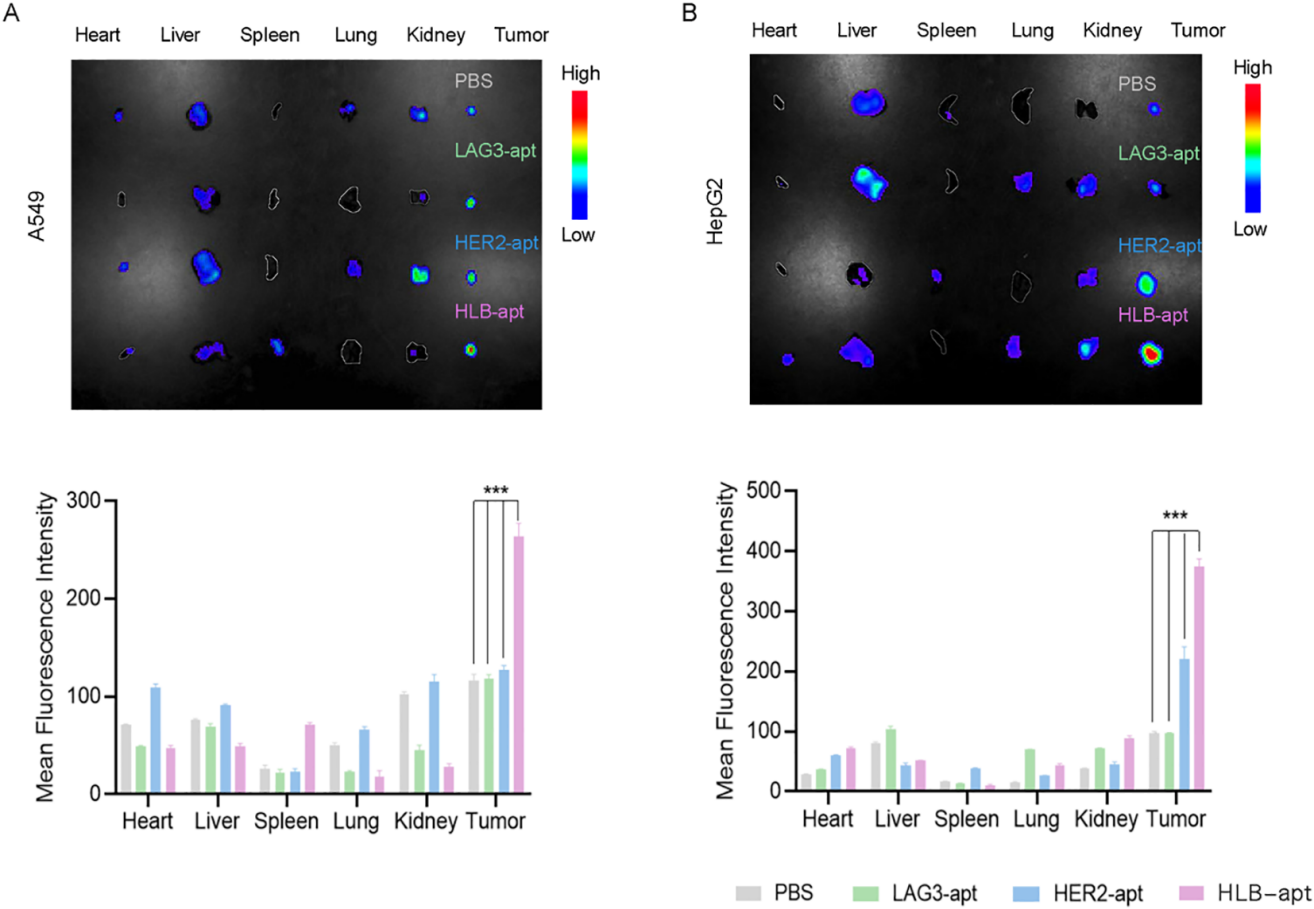
.Imaging distribution of HLB-apt in mice. **(A)** In vivo biological distribution of different aptamers in A549 cancer bearing mice; **(B)** In vivo biological distribution of different aptamers in HepG2 cancer bearing mice. (Data analysis was conducted by student t-test (comparison between two groups) or one-way analysis of variance (comparison among multiple groups), and the significance levels were ***P<0.001.)

To further study the biological distribution of HLB-apt *in vivo* in tumor-bearing nude mice. When the tumor volume of A549 tumor bearing mice and HepG2 tumor bearing mice reached a certain size, they were divided into four groups: PBS, LAG3-apt with cy5 fluorescence label, HER2-apt with cy5 fluorescence label and HLB-apt with cy7 fluorescence label. They were then injected into A549 and HepG2 tumor-bearing mice via the tail vein, respectively. After 24 h, the major organs (heart, liver, spleen, lungs, kidneys) and tumors were dissected. As shown in **Figure 6**, the fluorescence intensity of tumor tissue in HLB-apt and HER2-apt treatment groups was significantly higher than that in LAG3-apt and PBS treatment groups (*P<*0.001). More importantly, the fluorescence intensity of the HLB-apt treatment group was higher than that of the HER2-apt treatment group, indicating that HLB-apt could bind to tumor cells better than the HER2-apt treatment group. This is because both HER2-apt and HLB-apt can specifically bind HER2, which is highly expressed in tumor cells (A549, HepG2 cells).

### Anticancer effects of HLB-apt and Jurkat cells *in vivo*

**Figure 7:**
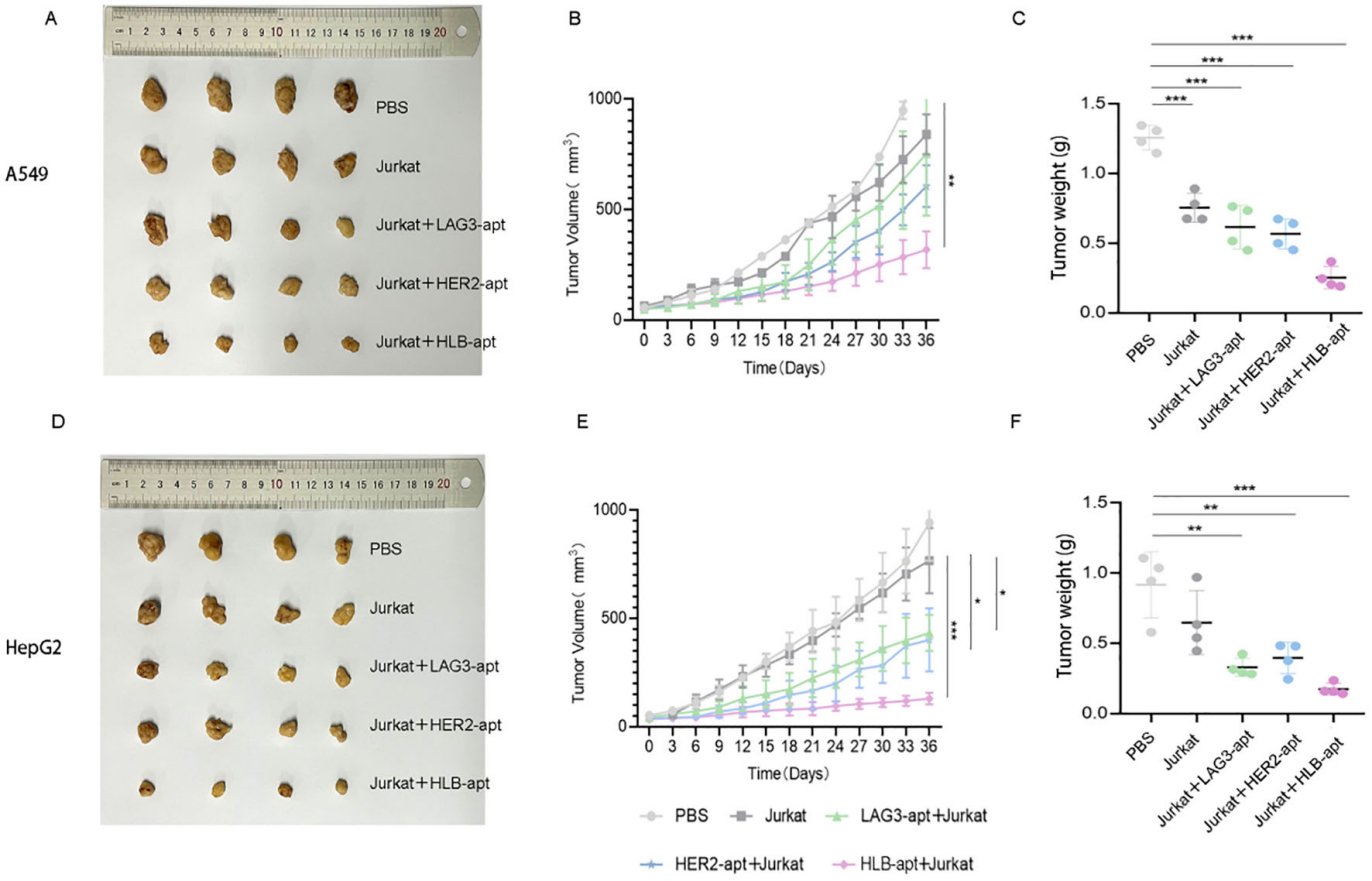
*In vivo* anti-tumor effects of HLB-apt and Jurkat cells. **(A)** After different treatments, A549 tumor bearing mice were photographed; **(B)** After different, tumor volume statistics were performed on A549 tumor bearing mice **(C)** After different treatments, tumor weight statistics were performed on A549 tumor bearing mice; **(D)** After different treatments, HepG2 tumor bearing mice were photographed; **(E)** After different, tumor volume statistics were performed on HepG2 tumor bearing mice; **(F)** After different treatments, tumor weight statistics were performed on HepG2 tumor bearing mice. (Data analysis was conducted by student t-test (comparison between two groups) or one-way analysis of variance (comparison among multiple groups), and the significance levels were *P<0.05, **P<0.01, and ***P<0.001).

To investigate whether HLB-apt and Jurkat cells can inhibit tumor growth in mice, we constructed A549 and HepG2 tumor bearing mouse models, respectively. When the tumor volume of the mice reached about 50 mm^3^, PBS, Jurkat cells, LAG3-apt + Jurkat cells, HER2-apt + Jurkat cells, and HLB-apt + Jurkat cells were used for treatment, and the mice were euthanized 36 days later. In A549 tumor bearing mice, the results showed that compared with the PBS group, treatment with HLB-apt+Jurkat cells resulted in a significant reduction in tumor weight in mice (1.256 g vs 0.252 g). In addition, the average volume of tumors in the PBS group was 1450.08mm^3^, the Jurkat cell group was 839.23mm^3^, the LAG3-apt + Jurkat cell group was 752.50mm^3^, the HER2-apt + Jurkat cell group was 605.47mm^3^, and the HLB-apt + Jurkat cell group was 318.23mm^3^. Compared with the PBS group, the tumor volume of mice in the HLB-apt + Jurkat cell group was significantly reduced (*P*<0.01)(**Figure 7**).

In HepG2 tumor bearing mice, the results showed that compared with the PBS group, HLB-apt + Jurkat cell therapy significantly reduced the tumor weight of mice (0.917 g vs 0.175 g). In addition, the average volume of tumors in the PBS group was 941.46mm^3^, the Jurkat cell group was 765.83mm^3^, the LAG3-apt + Jurkat cell group was 433.16 mm^3^, the HER2-apt + Jurkat cell group was 400.60 mm^3^, and the HLB-apt + Jurkat cell group was 129.83mm^3^. Compared with the PBS group, the tumor volume of mice in the HLB-apt + Jurkat cell group was significantly reduced (*P<*0.001)(**Figure 7**).

### Histopathological assessment of major organs

Finally, we also analyzed the potential toxicity of HLB-apt *in vivo.* After euthanizing the mice, they were dissected and their major organs (heart, liver, spleen, lungs, kidneys) and tumors were placed in 4% paraformaldehyde, followed by H&E staining.

**Figure 8:**
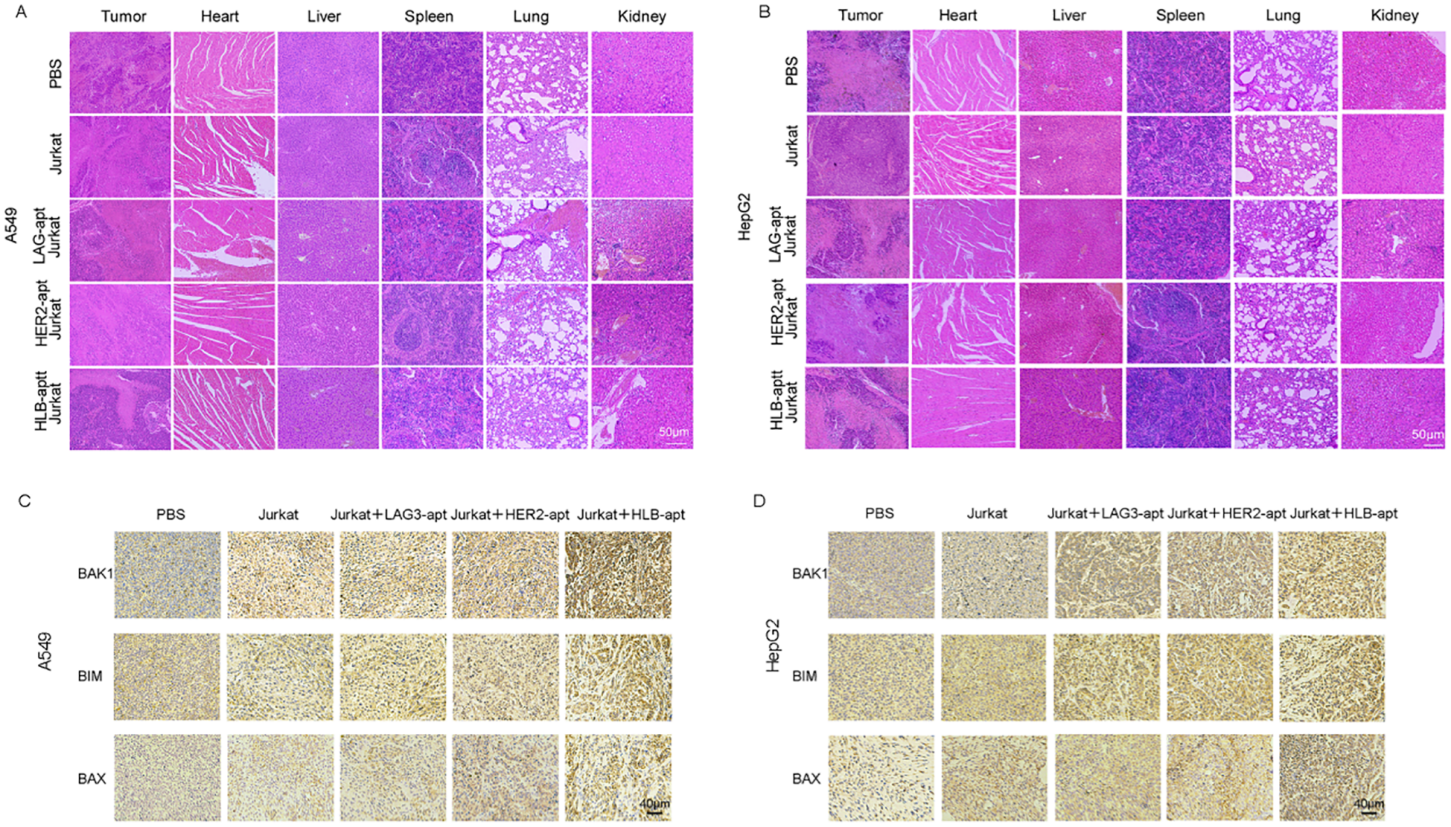
Histopathological assessment of major organs. **(A)** H&E staining of major organs and tumors in A549 tumor bearing mice after receiving different treatments; **(B)** H&E staining of major organs and tumors in HepG2 tumor bearing mice after receiving different treatments; **(C)** The expression of BAK1, BIM, and BAX in A549 mouse tumors after different treatments and immunohistochemistry; **(D)** The expression of BAK1, BIM, and BAX in HepG2 mouse tumors after different treatments and immunohistochemistry.

For A549, HepG2 tumor bearing mice, H&E images showed that compared with the PBS treatment group, there was no significant change in the HLB-apt treatment group, and there were no significant histological differences between different groups (**Figures 8A, B**). Therefore, this indicates that there are no significant histopathological abnormalities in the major organs of HLB apt treated mice.

Previous *in vitro* experiments have shown that HLB-apt induces apoptosis by upregulating the expression of BAK1, BIM, and BAX proteins in A549 and HepG2 cells. Furthermore, we further investigated the overexpression of BAK1, BIM, and BAX in tumors after different treatments through immunohistochemistry. Compared with other groups, simultaneous treatment of HLB-apt and Jurkat cells significantly upregulated the expression of BAK1, BIM, and BAX (**Figures 8C, D**).

## Discussion

In recent years, cancer immunotherapy has achieved good results in the field of cancer treatment, such as CAR-T therapy and ICIs. Although CAR-T therapy has shown good efficacy in CD19 positive malignant cancers (33), its widespread application in clinical practice is limited by certain factors, including antigen escape, off target phenomena, and lymphokine release syndrome (34, 35). In addition, The FDA has approved PD-1/PD-L1 inhibitors for the treatment of broad-spectrum solid cancers, but in clinical practice, The efficacy of PD-1/PD-L1 inhibitor monotherapy in unselected patients is not ideal (36, 37). However, they did not find effective anti-cancer immune responses in all cancer patients. Therefore, there is an urgent need to develop new cancer treatment methods. This article designed and constructed a HLB-apt that simultaneously targets HER2 and LAG3, in order to study its anti-cancer effects *in vitro* and *in vivo*.

HER2 is not only overexpressed in 20-30% of breast cancer, but also found in lung cancer, liver cancer, ovarian cancer and other cancers (19–23), and can be used as targets for targeted cancer treatment of most malignant cancers. Trastuzumab is the first approved HER2 monoclonal antibody drug, which has been used to treat metastatic HER2 positive breast cancer for more than ten years. However, the cancer targeting ability of monoclonal antibodies is often limited due to their insufficient penetration into cancer tissue. Therefore, it is necessary to explore other targeted treatment strategies for HER2. Compared with antibodies, aptamers have better cancer tissue permeability (38). In addition, aptamers have the characteristics of easy synthesis and modification, low immunogenicity, high stability, high targeting effect and low molecular weight (39–41), which make them attractive for cancer diagnosis and treatment. There have been many reports about targeted therapy of breast cancer with HER2-apt. The data of this study showed that the proliferation inhibition rates of A549 and HepG2 cells treated with HER2-apt for 48 h were 14.13% and 7.98% respectively, while the inhibition rates of the HLB-apt treatment group significantly increased to 18.81% and 34.01%. Further mechanism studies have shown that HLB-apt can effectively induce apoptosis in A549 and HepG2 cells by up-regulating the expression levels of pro-apoptotic proteins BAK1, BIM and BAX. However, the expression of caspase was not detected in this experiment. It is a key marker of apoptosis, which requires further exploration in the future. Xue (42) et al. developed a divalent HER2 aptamer-EGFR siRNA chimera, which can interfere with the functions of HER2 and EGFR receptors and induce apoptosis of HER2-positive breast cancer cells. Specifically, in SKBR3 cells, compared with the untreated group, 48 h of HER2-apt treatment increased the apoptosis/necrosis rate by 11.5%, while the chimeric treatment group increased it by 18.72%. Similarly, in BT474 cells, the HER2-apt group only increased cell death by 3.91%, while the chimeric treatment group increased by 12.19%. Our research shows that in A549 cells, the killing rate of the control group was 7.84%, that of the HER2-apt group was 9.88%, and that of the HLB-apt group was 29.00%. In the HepG2 group, the killing rate of the control group was 3.41%, that of the HER2-apt group was 4.65%, and that of the HLB-apt group was 7.46%. Compared with the research of Xu et al., HLB-apt has a better effect in A549 cells. The discrepancies between different cells might result from the discrepant expression of HER2.

LAG3 is a common cancer immunotherapy target expressed on activated human T cells and NK cells (14). Studies have demonstrated that LAG-3 plays a crucial role in regulating the expansion of activated T cells (43). Additionally, other research has indicated that LAG-3 is frequently co-expressed with various inhibitory receptors. This co-expression results in the down-regulation of cytosolic effector molecules (such as Gzms B and perforin) and effector cytokines (including IFN-γ and TNF-α) in CD8^+^ T cells. Moreover, key cytokines secreted by CD4^+^ T cells, such as IL-2, IL-4, and IFN-γ, are also diminished (44, 45). This study confirmed that both LAG3-apt and HLB-apt can promote the proliferation of Jurkat cells and up-regulate the expression levels of T-cell activation markers (CD69, CD3E) and cytokines (IL-2). Furthermore, it was found through ELISA detection that in the co-culture system with the two tumor cells, the levels of IL-2, TNF-α and Gzms B secreted by the HLB-apt group were significantly higher than those of the other treatment groups and the control group. Studies have shown that when LAG3 antibody is used to treat tumor-bearing mice with colorectal cancer MC38 and fibrosarcoma Sa1N, tumor growth slightly decreases and the clearance rate is very limited. Interestingly, in the MC38 tumor model, LAG3/PD1 blockade resulted in 80% of the animals being tumor-free. In the Sa1N tumor model, LAG3/PD1 blockade resulted in 70% of the animals being tumor-free (46). We have similar experimental results. In the A549 tumor-bearing mouse model, the tumor volumes of different treatment groups show significant differences: The average volume of the PBS control group was 1450.08 mm³, that of the Jurkat cell group was 839.23 mm³, that of the LAG3-apt + Jurkat group was 752.50 mm³, and that of the HER2-apt + Jurkat group was 605.47 mm³. The tumor volume in the HLB-apt + Jurkat group was the smallest (318.23 mm³), which was significantly less than that in the PBS group. A similar trend was also observed in the HepG2 tumor-bearing model: The tumor volume in the PBS group was 941.46 mm³, that in the Jurkat cell group was 765.83 mm³, and that in the LAG3-apt + Jurkat group and the HER2-apt + Jurkat group decreased to 433.16 mm³ and 400.60 mm³, respectively. The HLB-apt + Jurkat group showed the strongest inhibitory effect (129.83 mm³). This indicated that HLB-apt combined with Jurkat cells can effectively inhibit tumor growth in both tumor models.

Nevertheless, there remains significant potential for further exploration regarding the mechanisms underlying HLB-apt’s action. In particular, it is still an open question whether the immune-enhancing effects of HLB-apt are mediated through the blockade of inhibitory signal transduction pathways associated with LAG-3 or if they primarily function as a molecular bridge physically connecting Jurkat cells to tumor cells. This aspect warrants additional investigation in future studies. In addition, this study mainly focuses on the targeting effect of HLB-apt on tumor cells with high expression of HER2, while its binding characteristics in tumor cells with low expression of HER2 and normal tissues have not been fully clarified. Future studies need to systematically evaluate the tissue specificity of HLB-apt and its potential off-target effects, which is crucial for the safety assessment of clinical translation. In particular, by comparing the binding affinity and specificity of HLB-apt in cell lines with various HER2 expression levels, including primary normal cells, it will help to evaluate its therapeutic window more comprehensively. Existing studies have shown that in the mouse soft tissue sarcoma model, antibody blockade of LAG-3 can significantly inhibit tumor growth and increase the secretion level of IFN-γ in CD8^+^ cytotoxic T cells and CD4^+^ helper T cells (47). It is worth noting that in the *in vivo* experiments, this study did not systematically assess the risk of cytokine release syndrome that HLB-apt might cause. Although aptamer drugs usually have lower immunogenicity, their immune activation characteristics still need to be comprehensively evaluated before future clinical transformation.

In summary, we constructed HLB-apt by connecting HER2-apt and LAG3-apt through 10 base T connections. The HLB-apt has high affinity for HER2 positive cancer cells and Jurkat cells, tying the two types of cells together and significantly enhancing anti-cancer effects *in vitro* and *in vivo*, Specifically, HLB-apt significantly inhibited the growth of transplanted cancers in mice compared with the control group. The results indicate that HLB-apt based on LAG3/HER2 may serve as a novel method for selectively enhancing anti-cancer immune response.

## Conclusion

In summary, a novel HLB-apt based on LAG3-HER2 was developed for cancer immunotherapy. HLB-apt can recruit more Jurkat cells around cancer cells and activate Jurkat cells to secrete more cytokines. HLB-apt recruited Jurkat cells to HER2 overexpressing tumors through bispecific binding, which may concurrently disrupt immune checkpoint interactions. *In vivo* experiments demonstrated that HLB-apt can effectively localize around cancer cells, and its co-administration with Jurkat cells significantly inhibited tumor growth in tumor-bearing mice without causing observable tissue damage in histopathological analysis. Interestingly, there was a significant difference in the killing efficiency of HLB apt on A549 and HepG2 cells in this study, and we speculate that HER2 expression may be a key factor leading to the efficiency difference. Future research will combine single-cell sequencing and proteomics techniques to systematically analyze the correlation between target expression and therapeutic response. In brief, this study provides a promising strategy for immunotherapy of malignant cancers with high expression of HER2.

## Data Availability

The original contributions presented in the study are included in the article/supplementary material. Further inquiries can be directed to the corresponding author.
